# Supplementary material for: High Chern numbers in a perovskite-derived dice lattice (LaXO3)3/(LaAlO3)3(111) with X = Ti, Mn and Co
Source: Sci Rep. 2023 Jun 30;13:10615. doi: 10.1038/s41598-023-36170-9 (PMC10313702; doi:10.1038/s41598-023-36170-9)
Supplement: Supplementary file 1 — Supplementary Information. [file 41598_2023_36170_MOESM1_ESM.pdf]

Supplemental material: Electronic and topological properties of perovskite-derived dice lattices $(LaXO_3)_3/(LaAlO_3)_3(111)$ (X = Ti, Mn and Co)

Okan Köksal,[1] L. L. Li,[1] and Rossitza Pentcheva[1,*]

[1] *Department of Physics and Center for Nanointegration Duisburg-Essen (CENIDE), University of Duisburg-Essen, Lotharstr. 1, 47057 Duisburg, Germany*

(Dated: June 23, 2023)

COMPARISON BETWEEN FERRO- AND ANTIFERROMAGNETIC COUPLING

In Table S1 we compare the energies and properties of $(LaXO_3)_3/(LaAlO_3)_3(111)$ SLs (X = Ti, Mn and Co) for P3 symmetry with ferro- and (layerwise) antiferromagnetic coupling in the absence of SOC. For X = Ti, Mn and Co the antiferromagnetic arrangements are energetically less favorable by 0.19 eV, 0.87 eV and 1.10 eV than the respective ferromagnetic ground state.

TABLE S1. Comparison of structural, magnetic, and electronic properties between ferromagnetic and antiferromagnetic $(LaXO_3)_3/(LaAlO_3)_3(111)$ SLs (X = Ti, Mn and Co) for P3 symmetry in the absence of SOC. $\Delta E = E_{AFM} - E_{FM}$ is the energy difference between antiferromagnetic (AFM) configuration with respect to the ferromagnetic (FM) ground state, c is the optimized out-of-plane lattice constant, $d_{X\text{-O}}$ (IF/C) the X-O bond lengths in Å in the interfacial/central XO_6 octahedra, E_g the band gap in eV, and M_S the layer-resolved spin magnetic moments (in units of μ_B) at the X sites, IF1/IF2 denote the first/second interface layer and C the central one.

X	Magn. Order	ΔE (eV)	c (Å)	$d_{X\text{-O}}$ (IF)	$d_{X\text{-O}}$ (C)	E_g (eV)	M_S (IF1/C/IF2)
Ti	AFM	0.19	14.10	2.09	1.94	Metal	0.35/-1.63/0.35
	FM	0	14.23	2.06-2.07	1.96	Metal	1.32/0.22/1.32
Mn	AFM	0.87	13.80	2.04	1.88	Metal	4.31/-2.75/4.31
	FM	0	13.78	1.96-2.00	1.96	Metal	4.04/3.99/4.04
Co	AFM	1.10	13.64	1.99	1.92	Metal	2.32/-3.24/2.32
	FM	0	13.58	1.97-1.98	1.91	Metal	2.44/2.83/2.44

In Table S2 the results for the antiferromagnetic order with different symmetries are shown. Similar to the ferromagnetic solutions, the obtained energies for the P1 symmetry demonstrate that these are favored by 2.76 eV, 1.23 eV and 1.57 eV for X = Ti, Mn and Co, respectively. It is noteworthy that the symmetry reduction from P3 to P1 is accompanied by a substantial band gap opening of 2.38 eV for X = Ti and 1.46 eV for X = Mn and Co whereas the cases with P3 symmetry remain metallic.

TABLE S2. Structural, magnetic, and electronic properties of antiferromagnetic $(LaXO_3)_3/(LaAlO_3)_3(111)$ SLs (X = Ti, Mn and Co) for P3 and P1 symmetries in the absence of SOC. ΔE is the energy difference of the system in P3 symmetry compared to P1, c is the optimized out-of-plane lattice constant, $d_{X\text{-O}}$ (IF/C) the X-O bond lengths in Å in the interfacial/central XO_6 octahedra, E_g the band gap in eV, and M_S the layer-resolved spin magnetic moments (in units of μ_B) at the X sites, IF1/IF2 denote the first/second interface layer and C the central one.

X	Symmetry	ΔE (eV)	c (Å)	$d_{X\text{-O}}$ (IF)	$d_{X\text{-O}}$ (C)	E_g (eV)	M_S (IF1/C/IF2)
Ti	P3	2.76	14.10	2.09	1.94	Metal	0.35/-1.63/0.35
	P1	0	14.34	2.01-2.14	2.04-2.09	2.38	-0.97/0.96/-0.97
Mn	P3	1.23	13.80	2.04	1.88	Metal	4.31/-2.75/4.31
	P1	0	14.14	1.93-2.22	1.89-2.08	1.46	3.85/-3.85/3.85
Co	P3	1.57	13.64	1.99	1.92	Metal	2.32/-3.24/2.32
	P1	0	13.77	1.94-2.03	1.93-1.99	1.46	3.10/-3.02/3.10

DFT+U+SOC RESULTS FOR P1 SYMMETRY

In the following, the effect of SOC and topological analysis in $(LaXO_3)_3/(LaAlO_3)_3(111)$ SLs with X = Ti, Mn and Co and lowered P1 symmetry are presented. The corresponding DFT+U+SOC band structures are displayed in Figures S1, S2 and S3 which show in all cases a Mott insulating ground state and substantial band gaps of 2.34 eV, 0.40 eV and 0.44 eV for X = Ti, Mn and Co, respectively. In contrast to the cases with P3 symmetry, the inclusion of SOC does not result in a significant band reconstruction for both in-plane and out-of-plane magnetization directions.

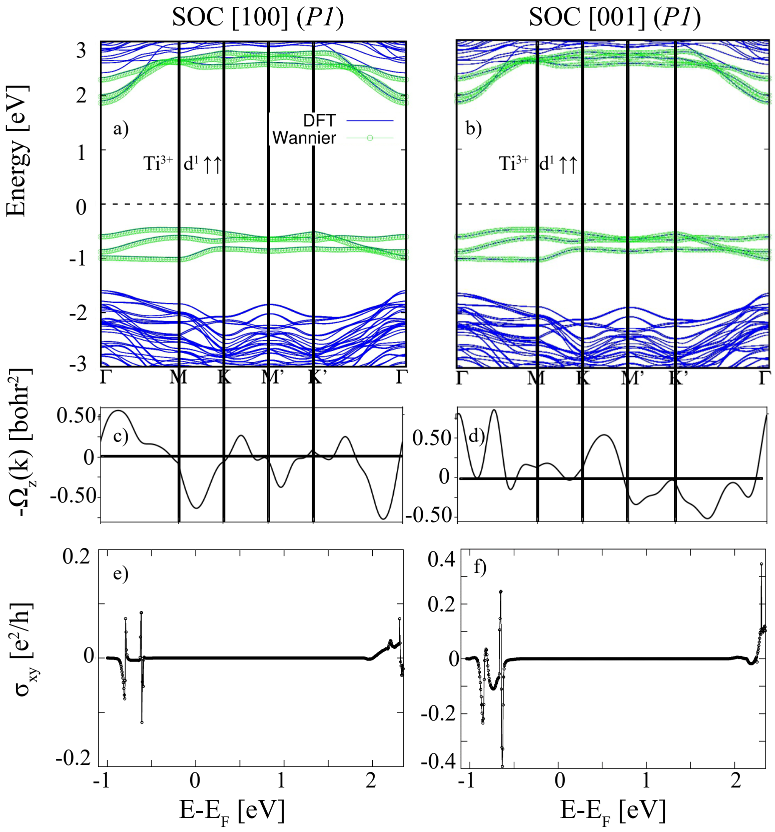

FIG. S1. a-b) Comparison between the GGA+U+SOC band structures (blue lines) and the Wannier interpolation (dotted green lines) for X = Ti with P1 symmetry for magnetization directions along [100] and [001] as well as c-d) the Berry curvatures $\Omega_{xy}(k)$ along the same k-path. e-f) show the corresponding anomalous Hall conductivities $\sigma_{xy}^{\mathrm{AHC}}$ vs. the chemical potential in units of e^2/h.

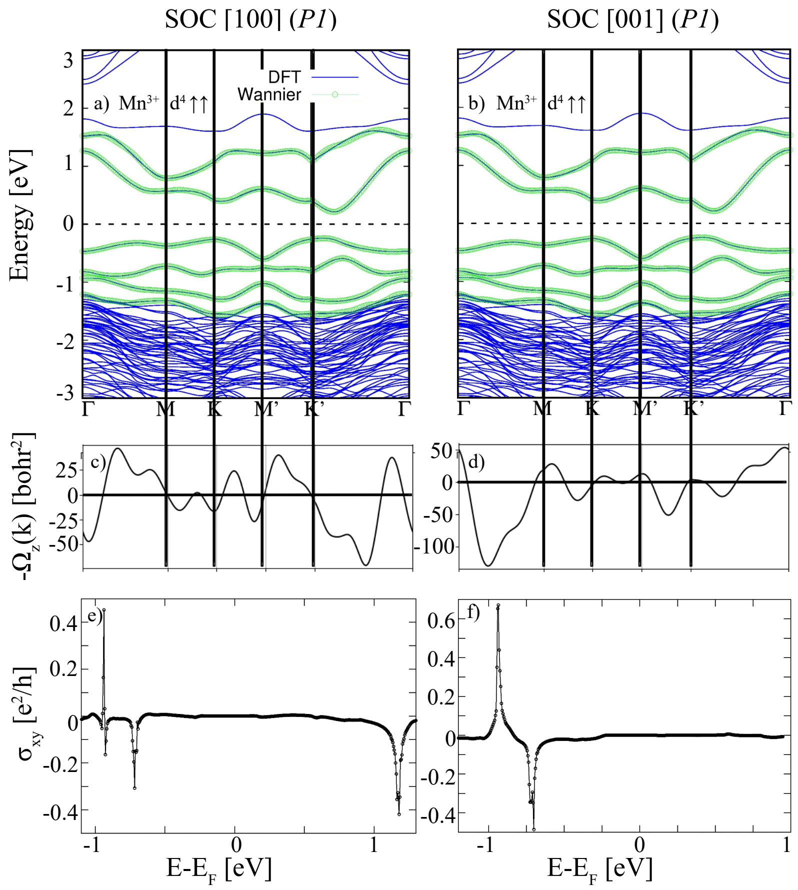

FIG. S2. a-b) Comparison between the GGA+U+SOC band structures (blue lines) and the Wannier interpolation (green dotted lines) for X = Mn with P1 symmetry for magnetization directions along [100] and [001] as well as c-d) the Berry curvatures $\Omega_{xy}(k)$ along the same k-path. e-f) show the corresponding anomalous Hall conductivities $\sigma_{xy}^{\mathrm{AHC}}$ vs. the chemical potential in units of e^2/h.

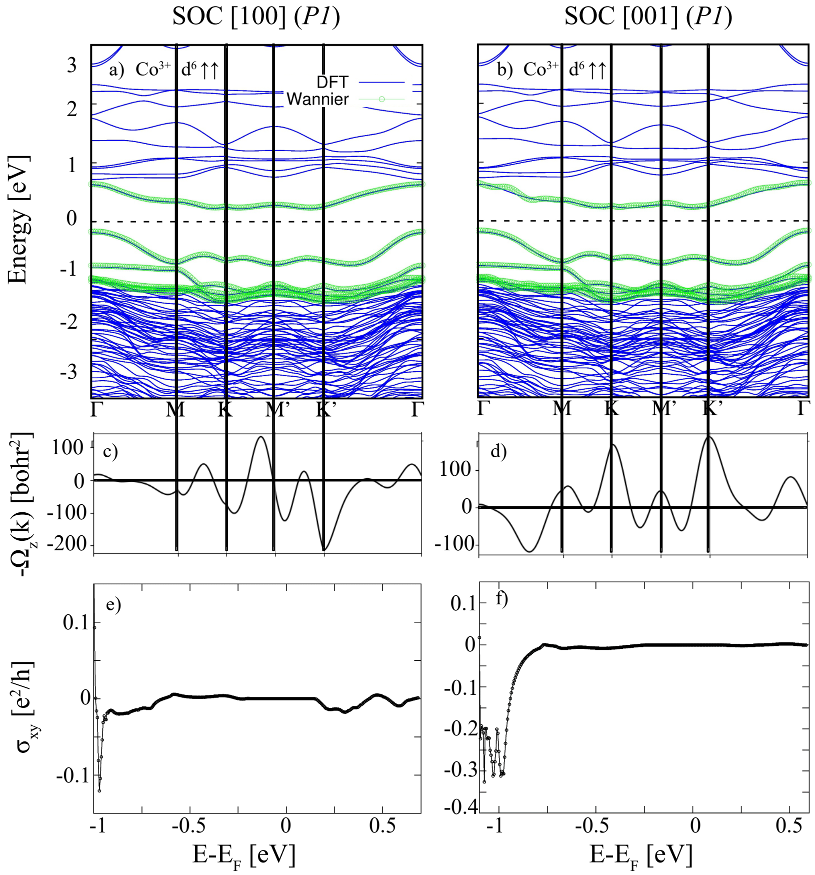

FIG. S3. a-b) Comparison between the GGA+U+SOC band structures (blue lines) and the Wannier interpolation (dotted green lines) for X = Co with P1 symmetry for magnetization directions along [100] and [001] as well as the Berry curvatures $\Omega_{xy}(k)$ c-d) along the same k-path. e-f) show the corresponding anomalous Hall conductivities $\sigma_{xy}^{\mathrm{AHC}}$ vs. the chemical potential in units of e^2/h.

COMPARISON BETWEEN THE GGA+U+SOC BAND STRUCTURES AND THE WANNIER INTERPOLATION WITH P3 SYMMETRY

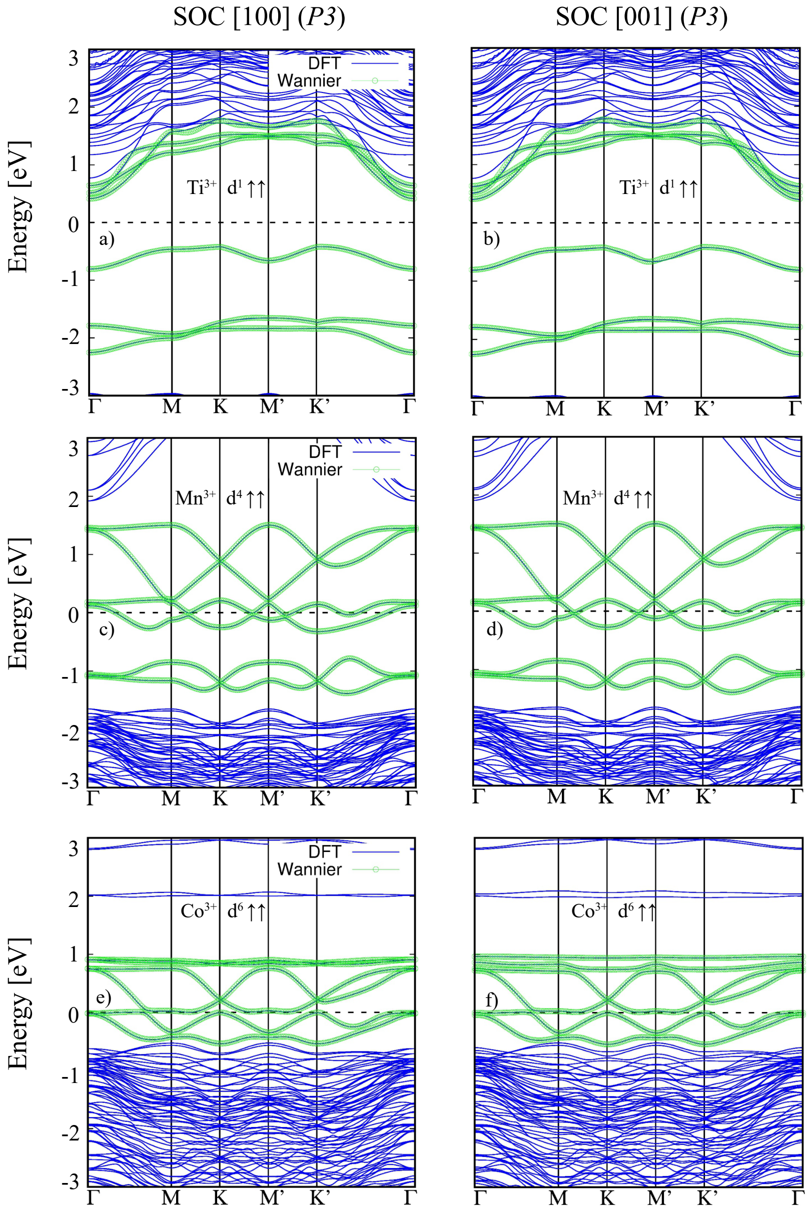

FIG. S4. Comparison between the GGA+U+SOC band structures of a-b) X = Ti, c-d) X = Mn and e-f) X = Co with P3 symmetry calculated by DFT (blue line) and the one obtained by Wannier interpolation (dotted green lines) for magnetization directions along [100] and [001], respectively.

In Fig. S4 the quality of Wannier fit for X = Ti, Mn and Co in P3 symmetry is demonstrated by superimposing the Wannier-interpolated bands on the original DFT +U+SOC bands with energy windows X = Ti [−2.3 eV, 1.8 eV], X = Mn [−1.4 eV, 1.6 eV] and X = Co [−0.5 eV, 1.0 eV], incliding the X 3d bands and O 2p bands.

DEPENDENCE OF THE TOPOLOGICAL PROPERTIES ON THE HUBBARD U PARAMETER

Additionally, we explored the effect of the U parameter on the band structure and topological properties for X = Mn in P3 symmetry and [001] magnetization direction (see Fig. S5). While the overall band structure around the

Fermi level persists for all studied values, the overlap with other unoccupied bands for $U = 0.5$ eV and $U = 1.0$ eV quenches the high Chern numbers around E_F (cf. S5a-b). The emergence of high Chern numbers is robust beyond $U = 3.0$ eV up to the maximum value of 6 eV considered here.

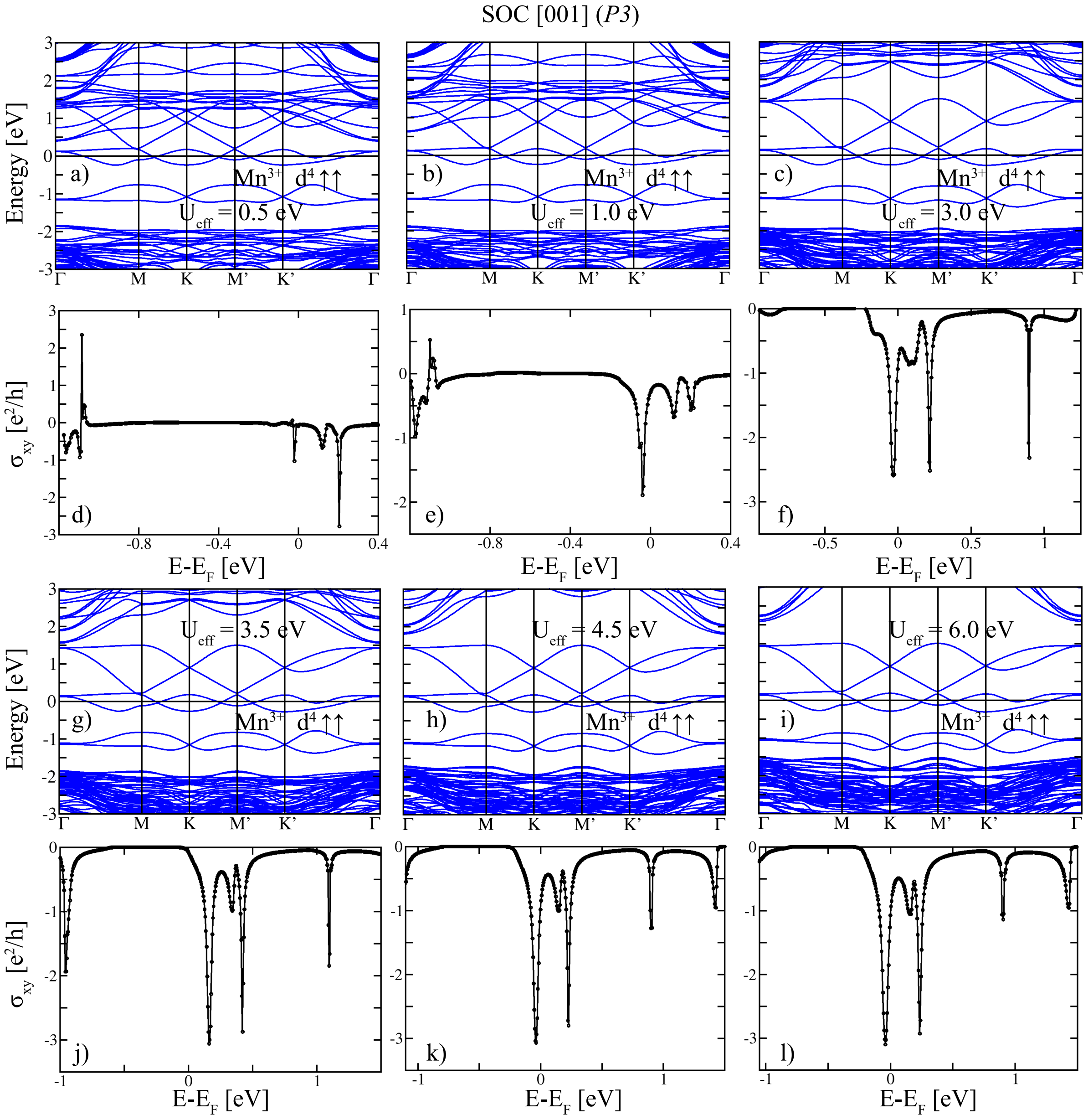

FIG. S5. GGA + U + SOC results with magnetization direction along [001] for X = Mn as a function of the on-site Coulomb repulsion parameter U: Evolution of the band structure (a-c, g-i) and the corresponding anomalous Hall conductivity σ_{xy} in units of e^2/h vs. the chemical potential (d-f, j-l).

EFFECT OF STRAIN

Furthermore, we investigated the effect of strain on the relative stability of the P3 and P1 phases for $X =$ Mn. We find that the energy vs. lateral lattice parameter curves for P3 and P1 symmetry cross and P3 is slightly more favorable between $a = 4.04$ Å and $a = 4.14$ Å (see Fig. S6). We have also analyzed the topological properties of both P1 and P3 at $a = 4.04$ Å (cf. Fig. S7).

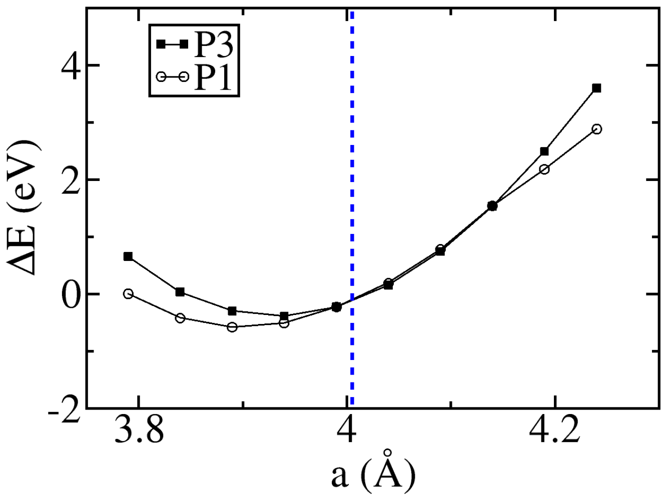

FIG. S6. Energy difference w.r.t. the ground state P1 symmetry per u.c. vs. in-plane lattice constant for $X = \text{Mn}$ (circle and square symbols denote P1 and P3 symmetry). For each value of the lateral strain, the out-of-plane lattice constant was optimized within GGA+U.

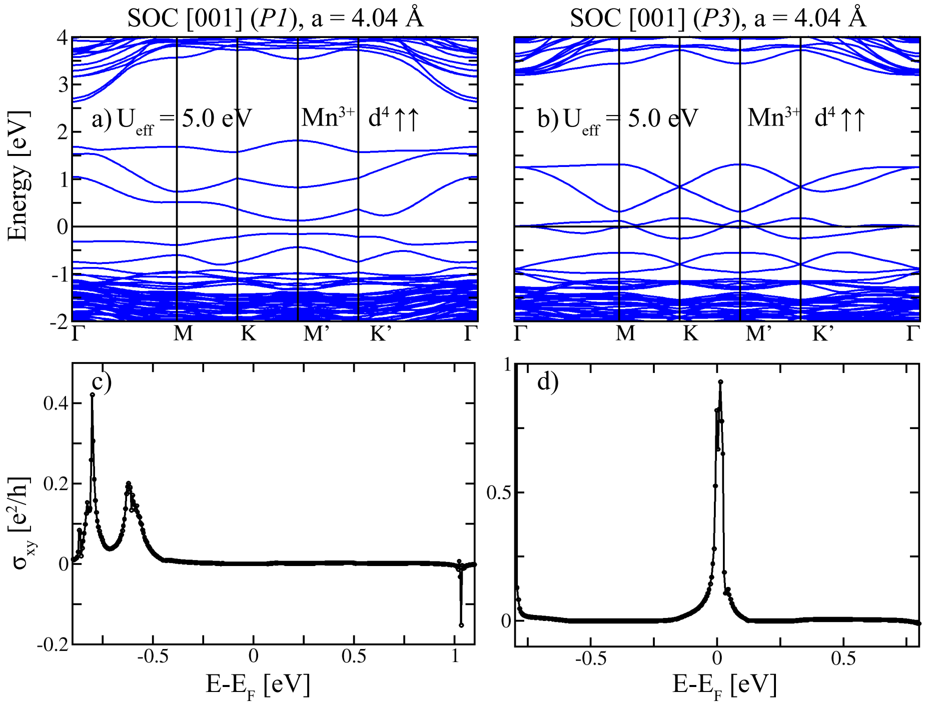

FIG. S7. GGA + U + SOC band structures for $X = \text{Mn}$ at a = 4.04 Å for (a) P1 and (b) P3 symmetry, respectively. c-d) show the corresponding anomalous Hall conductivities σ_{xy}^{AHC} as a function of the chemical potential in units of e^2/h.

The GGA + U + SOC band structure for $X = \text{Mn}$ at a = 4.04 Å with P1 symmetry in Fig. S7a shows that the relevant bands around E_{F} get more disentangled under tensile strain than at a_{LAO} (cf. Fig. S2b). For P3 symmetry the bands just above the Fermi level are shifted upwards and disentangled from the two bands at E_{F} under tensile strain S7b) compared to the case at a_{LAO} (cf. Fig. S4d). The anomalous Hall conductivity σ_{xy} for P3 symmetry exhibits a significant, almost integer value of $\sim 0.94e^2/h$ (see Fig. S7d).

* rossitza.pentcheva@uni-due.de
